# Correction to: Primary adrenal lymphoma: three case reports and review of Japanese cases

**DOI:** 10.1007/s13691-018-0343-0

**Published:** 2018-09-01

**Authors:** Shigeaki Nakazawa, Motohide Uemura, Takeshi Ujike, Kazutoshi Fujita, Tetsuya Takao, Yasushi Miyagawa, Norio Nonomura

**Affiliations:** Department of Urology, Osaka University Graduate School of Medicine, 2-2 Yamadaoka, Suita City, Osaka 565-0871 Japan

## Correction to: Int Canc Conf J (2015) 4:195–200 10.1007/s13691-014-0198-y

The article Primary adrenal lymphoma: three case reports and review of Japanese cases, written by Shigeaki Nakazawa, Motohide Uemura, Takeshi Ujike, Kazutoshi Fujita, Tetsuya Takao, Yasushi Miyagawa and Norio Nonomura, was originally published electronically on the publisher’s internet portal (currently SpringerLink) on 4 November 2014 without open access.

With the author(s)’ decision to opt for Open Choice the copyright of the article changed on 5 September 2018 to © The Author(s) 2018 and the article is forthwith distributed under the terms of the Creative Commons Attribution 4.0 International License (http://creativecommons.org/licenses/by/4.0/), which permits use, duplication, adaptation, distribution and reproduction in any medium or format, as long as you give appropriate credit to the original author(s) and the source, provide a link to the Creative Commons license and indicate if changes were made.

